# Reply to Paredes et al. Comment on “Aaseth et al. Circulating Lipoproteins in Subjects with Morbid Obesity Undergoing Bariatric Surgery with Gastric Bypass or Sleeve Gastrectomy. *Nutrients* 2022, *14*, 2381”

**DOI:** 10.3390/nu15010053

**Published:** 2022-12-22

**Authors:** Jan O. Aaseth, Helge Rootwelt, Kjetil Retterstøl, Knut Hestad, Per G. Farup

**Affiliations:** 1 Department of Research, Innlandet Hospital Trust, N-2381 Brumunddal, Norway; 2 Department of Health and Nursing Science, Faculty of Health and Social Sciences, Inland Norway University of Applied Sciences, N-2418 Elverum, Norway; 3 Department of Medical Biochemistry, Oslo University Hospital, N-0424 Oslo, Norway; 4 Department of Nutrition, Institute of Basic Medical Sciences, University of Oslo, N-0317 Oslo, Norway; 5 Lipid Clinic, Oslo University Hospital, N-0424 Oslo, Norway; 6 Department of Clinical and Molecular Medicine, Faculty of Medicine and Health Sciences, Norwegian University of Science and Technology, N-7491 Trondheim, Norway

Hereby, we thank Silvia Paredes et al. [1] for their important comments to our paper entitled “Circulating Lipoproteins in Subjects with Morbid Obesity Undergoing Bariatric Surgery with Gastric Bypass or Sleeve Gastrectomy” where changes in lipid profile in obese individuals after bariatric surgery were reported [2]. Cardiovascular disease remains the leading cause of death in developed countries, and it is well known that obese people with metabolic syndrome and dyslipidemia are particularly vulnerable. In our pre- and postoperative evaluation of the lipoproteins, we considered it of importance to include not only the traditional lipid components but also lipoprotein (a) and non-high-density cholesterol (non-HDL-c). As pointed out in the comments by Silvia Paredes et al., non-HDL-c has been recognized by the European Society of Cardiology and European Atherosclerosis Society to be one of the preferred lipoproteins to be measured in patients at risk, such as individuals with type 2 diabetes mellitus, metabolic syndrome, or high triglyceride levels [3].

In their research, Silvia Paredes et al. measured non-HDL-c in patients submitted to sleeve gastrectomy (SG, *n* = 185) and Roux-en-Y gastric bypass (RYGB, *n* = 18), preoperatively and 12 months after bariatric surgery. In their sample, they identified statistically significant decreases in non-HDL-c after surgery both in the SG and in the RYGB group, but larger differences for RYGB than for SG. Compared with our study, which identified a non-HDL-c decrease of about 15% in patients after RYGB, but no significant change after SG, they found a 28.5% and 10.3% non-HDL-c decrease, respectively, in patients submitted to the same procedures. From their results, it is also apparent that RYGB exerts a higher lipid lowering effect than SG; however, they also noticed the role of SG in reducing non-HDL-c. 

Many factors may contribute to the discrepancy between the two studies. Paredes et al. performed their analyses after excluding patients using lipid lowering drugs [1], while in our sample, two patients remained on such treatment [2]. Furthermore, Paredes et al. included only data collected 12 months after surgery, whereas in our study the data collected both at 6 and 12 months after surgery were included in the analysis. Before allocation to RYGB and SG, Paredes et al. [1] found mean non-HDL-c levels of 3.89 ± 0.86 mmol/L and 3.79 ± 0.84 mmol/L, respectively. However, they did not describe any preoperative lifestyle intervention. In our study, the participants went through an interventional lifestyle regimen for six months, with advice on dietary habits and physical activity. The regimen was further intensified three weeks before the surgery, when they followed a strict “crispbread diet” with a maximum daily intake of 4200 kJ [4]. During the preoperative intervention period, the average non-HDL-c was reduced from 3.90 mmol/L to 3.26 mmol/L. Thus, in our study, the impact of bariatric surgery on non-HDL-c (about 15% after RYGB and no significant change after SG) might be considered rather modest when compared with the results of preoperative lifestyle intervention, which led to a reduction of about 16% in non-HDL-c. This observation emphasizes the favourable effects of lifestyle intervention. Most probably, our strict preoperative lifestyle regimen represents the main contributing factor to the alleged discrepancy between the results presented by Silvia Paredes et al. and the results in our paper [2].

As for our study [2], we consider the observed significant reduction in lipoprotein (a) (about 26%) after RYGB to be the most intriguing observation. Surprisingly, this lipoprotein reduction appeared not to be related to weight reduction, and the mechanism behind this effect remains unknown. This observation may encourage new approaches to research on lipoprotein-lowering mechanisms. It is known that statins and other conventional lipid-lowering agents have minimal or no effects on lipoprotein (a) [5]. Lowering of lipoprotein (a) will contribute to the cardioprotective effect of the surgery.

Today, RYGB and SG are the most common bariatric procedures. The SG method has appeared to be associated with less malabsorption than RYGB [6], and thus associated with fewer adverse effects, which may have contributed to its increasing popularity in recent years. However, RYGB could allow for better disease management in patients with high cardiovascular risk. Further research and understanding of the importance of the surgical method for reducing cardiovascular risk is anticipated to lead to improvements in the surgical approach.
